# HIF-1α Mediates TRAIL-Induced Neuronal Apoptosis *via* Regulating DcR1 Expression Following Traumatic Brain Injury

**DOI:** 10.3389/fncel.2020.00192

**Published:** 2020-08-03

**Authors:** Yuanjian Fang, Jianan Lu, Xiaoyu Wang, Haijian Wu, Shuhao Mei, Jingwei Zheng, Shenbin Xu, Cameron Lenahan, Sheng Chen, Jianmin Zhang, Yuan Hong

**Affiliations:** ^1^Department of Neurosurgery, The Second Affiliated Hospital, School of Medicine, Zhejiang University, Hangzhou, China; ^2^Center for Neuroscience Research, Loma Linda University School of Medicine, Loma Linda, CA, United States; ^3^Burrell College of Osteopathic Medicine, Las Cruces, NM, United States; ^4^Brain Research Institute, Zhejiang University, Hangzhou, China; ^5^Collaborative Innovation Center for Brain Science, Zhejiang University, Hangzhou, China

**Keywords:** traumatic brain injury, tumor necrosis factor-related apoptosis-inducing ligand, death receptor 5, decoy receptor, hypoxia-induced factor-1α

## Abstract

**Background**: Neuronal apoptosis involved in secondary injury following traumatic brain injury (TBI) significantly contributes to the poor outcomes of patients with TBI. The tumor necrosis factor-related apoptosis-inducing ligand (TRAIL) can selectively induce apoptosis of tumor cells. Hypoxia factor (HIF) 1α is a controversial factor that mediates the neuronal apoptotic pathway. Herein, we hypothesize that HIF-1α may mediate the TRAIL-induced neuronal apoptosis after TBI.

**Methods**: We used Western blots and immunofluorescence to study the expression and cell localization of TRAIL and death receptor 5 (DR5) after TBI in rats. Soluble DR5 (sDR5) administration was used to block the TRAIL-induced neuronal death and neural deficits. HIF-1α inhibitor 2ME and agonist DMOG were used to study the role of HIF-1α in TRAIL-induced neuronal death. Meanwhile, HIF-1α siRNA was used to investigate the role of HIF-1α in TRAIL-induced neuronal death *in vitro*.

**Results**: The expressions of microglia-located TRAIL and neuron-located DR5 were significantly upregulated after TBI. sDR5 significantly attenuated TRAIL-induced neuronal apoptosis and neurological deficits. 2ME decreased neuronal apoptosis, lesion area, and brain edema and improved neurological function *via* increased expression of TRAIL decoy receptor 1 (DcR1), which inhibited TRAIL-induced apoptosis after TBI. The administration of DMOG produced the opposite effect than did 2ME. Similarly, HIF-1α siRNA attenuated TRAIL-induced neuronal death *via* increased DcR1 expression *in vitro*.

**Conclusion**: Our findings suggested that the TRAIL/DR5 signaling pathway plays an important role after neuronal apoptosis after TBI. HIF-1α mediates TRAIL-induced neuronal apoptosis by regulating DcR1 expression following TBI.

## Introduction

Traumatic brain injury (TBI) is one of the leading causes of death and disability in patients with trauma. Approximately 10 million people, especially those under 45 years old, have been reported to suffer TBI annually worldwide (Wu et al., 2016; Zhou et al., 2020). Currently, the detailed pathological mechanism of TBI remains unclear, and effective treatments are severely lacking. Therefore, in-depth studies regarding the pathological mechanisms, particularly emphasizing the cellular and molecular changes following TBI, are urgently needed. Secondary brain injury after TBI is the key factor in affecting the prognosis of patients. Neuronal damage caused by secondary brain injury is similar to cerebral ischemia-reperfusion injury. It is mutually promoted by various pathogenic mechanisms, including excitotoxicity, inflammatory response, oxidative stress, calcium overload, etc. The endpoint of various pathophysiological changes is the apoptosis of neuronal cells, which is the main cause of long-term dysfunction in patients after TBI (Stoica and Faden, 2010; Guo et al., 2016).

The tumor necrosis factor (TNF)-related apoptosis-inducing ligand (TRAIL), a member of the TNF family, can selectively induce apoptosis of tumor cells. It was found that the TRAIL-related signaling pathway may also be related to the pathophysiological change in non-neoplastic diseases, such as diabetes (Bossi et al., 2015), atherosclerosis (Michowitz et al., 2005), rheumatoid arthritis (Dessein et al., 2015), pulmonary hypertension (Lawrie, 2014), and viral hepatitis (Mundt et al., 2005). Furthermore, numerous studies have confirmed that TRAIL-related signaling pathways are involved in the pathology of a variety of central nervous system disorders, including Alzheimer’s disease (Wu et al., 2015), multiple sclerosis (Lopez-Gomez et al., 2016), and ischemic stroke (Cui et al., 2010). Elevated TRAIL expression in the brain after ischemic stroke could aggravate neuronal apoptosis and cause brain damage, leading to poor prognosis (Cui et al., 2010; Cantarella et al., 2014). In humans, four membrane-bound receptors for TRAIL have been identified. Of these, only two death receptors DR4 (TRAIL-R1) and DR5 (TRAIL-R2) are transmembrane proteins equipped with an intracellular death domain (DD), which activates caspase-dependent apoptotic cell death. There are two other decoy receptors, DcR1 (TRAIL-R3) and DcR2 (TRAIL-R4), but they are incapable of death signaling. DcR1 owns a truncated, non-functional DD, and DcR2 lacks an intracellular domain (Schneider et al., 2003; Hoffmann et al., 2009). In contrast, only one TRAIL receptor with death signaling capacity (DR5) and two decoy receptors was found in the murine system (Wu et al., 1999). Death receptors and decoy receptors competitively combined with TRAIL to mediate the apoptosis in mammal.

Meanwhile, previous studies have confirmed that ischemia and hypoxia can cause upregulation of hypoxia factor (HIF)-1α after TBI (Li et al., 2013). Interestingly, the effect of HIF-1α on the apoptotic pathway remains controversial, and it may show distinct pro- and anti-apoptotic effects under different pathological conditions. HIF-1α-knockout Chinese hamster ovary cells can tolerate apoptosis induced by hypoxia (Carmeliet et al., 1998). Additionally, Li et al. found that the expression of p53 was increased and neuronal apoptosis was attenuated by using HIF-1α siRNA in neuron (Li et al., 2013). HIF-1α inhibitor 2ME attenuated brain injury after TBI *via* the inhibition of a maladaptive HIF-1α-dependent response (Schaible et al., 2014). Conversely, there have also been many studies showing that HIF-1α has an anti-apoptotic effect (Erler et al., 2004; Greijer and van der Wall, 2004; Piret et al., 2005). It was found long-term hypoxic tumors are not sensitive to TRAIL treatment and that inhibition of HIF-1α expression increases tumor sensitivity to TRAIL and promotes apoptosis, suggesting that HIF-1α may regulate apoptosis through the TRAIL pathway (Jeong et al., 2010). A separate study further confirmed that this anti-apoptotic effect of HIF-1α was mediated by the inhibition of TRAIL receptor DcR2 (Pei et al., 2010). These results suggest that the effect of HIF-1α on the apoptotic pathway may not be unique, and it may exhibit two distinct pro- and anti-apoptotic effects under different pathological conditions.

Considering that TBI shares many similar pathophysiological processes with cerebral ischemia, we speculate that the TRAIL-induced apoptosis pathway may be involved in cell apoptosis after TBI and also be regulated by HIF-1α. Therefore, the aim of the current study was to investigate the expression pattern of the TRAIL apoptosis pathway following TBI and to evaluate whether HIF-1α is involved in neuronal apoptosis through regulation of the TRAIL pathway.

## Materials and Methods

### Study Design

**Figure 1:**
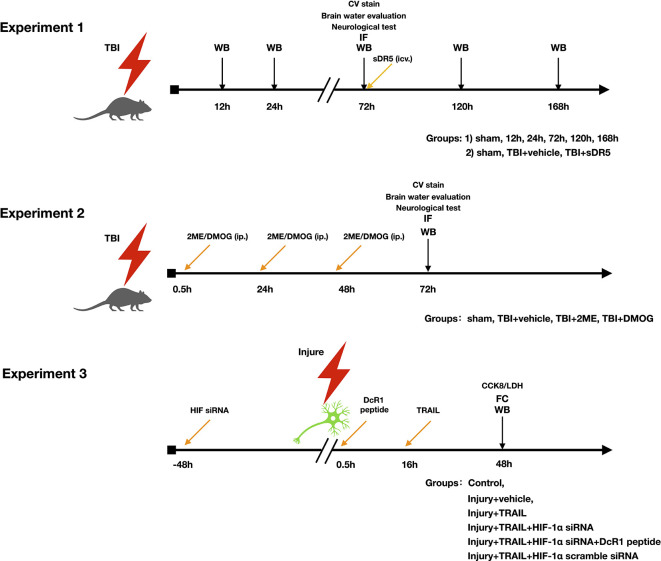
Experimental design and groups.

The study was divided into three parts (Figure 1). The objective of the first part was to investigate the role of the TRAIL signaling pathway in neuronal apoptosis after TBI. The expression of TRAIL and receptors DR5, DcR1, and DcR2 in the injured cortex were studied. The cell locations of TRAIL and DR5 in the injured cortex protein after TBI were also investigated. Soluble DR5 (sDR5) was used to block the TRAIL-induced apoptosis, which was detected by expression of cleaved caspase-3, by Fluoro-Jade C (FJC) staining, and by lesion area [Cresyl Violet (CV) staining].

The objective of the second part was to investigate the role of HIF-1α on the TRAIL-induced neuronal apoptosis pathway. Rats were divided into four groups: sham, TBI + vehicle, TBI + 2ME, and TBI + DMOG. DMOG and 2ME were injected intraperitoneally as HIF-1α inhibitor and activator, respectively. The expression of TRAIL and TRAIL receptors, including DR5, DcR1, and DcR2, were detected after treatment. HIF-1α-induced neuronal apoptosis was detected by expression of cleaved caspase-3, FJC staining, and lesion area.

The third part aimed to verify the role of HIF-1α in the TRAIL-induced neuronal apoptosis pathway in an *in vitro* model using the HT-22 cell line. Inhibition of HIF-1α was conducted using HIF-1α siRNA. Additionally, a DcR1 blocking peptide was given to inhibit the function of DcR1. Cells were divided into six groups: control, injury + vehicle, injury + TRAIL, injury + TRAIL + HIF-1α siRNA, injury + HIF-1α siRNA + DcR1 peptide, and TBI + HIF-1α scramble RNA. The effect of HIF-1α on DcR1 and DcR1-mediated apoptosis was investigated.

### Animals and Cell Line

Adult male Sprague Dawley rats weighing 280–320 g were purchased from SLAC Laboratory Animal Company Limited (Shanghai, China). All animal experiments were performed after receiving approval from the Institutional Ethics Committee of the Second Affiliated Hospital, Zhejiang University School of Medicine. The procedures were performed in compliance with the National Institutes of Health’s Guide for the Care and the Use of Laboratory Animals and the ARRIVE (Animal Research: Reporting *in vivo* Experiments) guidelines. The rats were housed in air-filtered temperature-controlled units with a 12-hour light/dark cycle. Rats were provided ad libitum access to food and water.

The murine hippocampal neuron cell line, HT-22, was cultured (37°C, 5% CO_2_) in Dulbecco’s modified Eagle’s medium (Thermo Fisher, Waltham, MA, USA) with 10% fetal bovine serum (Sigma-Aldrich, USA), 100 U/ml penicillin, and 100 μg/ml streptomycin.

### TBI Model

**Figure 2:**
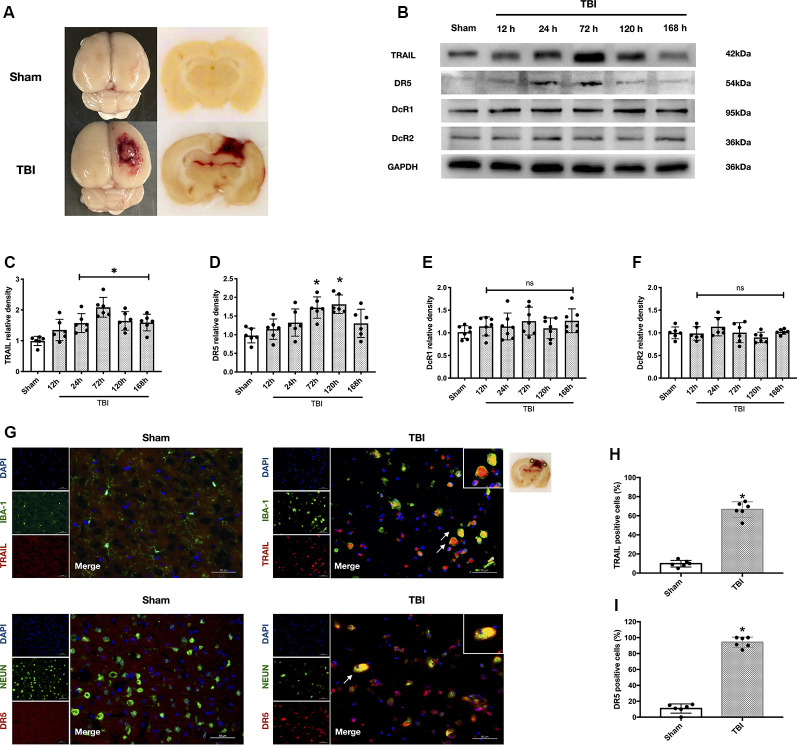
Protein expression level and cellular localization of tumor necrosis factor-related apoptosis-inducing ligand (TRAIL) and death receptor 5 (DR5). **(A)** Representative picture of the traumatic brain injury (TBI) model. **(B)** Representative Western blot bands of each protein. **(C)** Densitometric quantification of TRAIL. **(D)** Densitometric quantification of DR5. **(E)** Densitometric quantification of decoy receptor 1 (DcR1). **(F)** Densitometric quantification of DcR2. **(G)** Representative microphotographs of immunofluorescence staining showing localization of TRAIL and DR5 (red), Iba-1 and NeuN (green) in injured cerebral cortex after TBI. Scale bar = 50 μm. **(H)** Quantification of TRAIL-positive cells in sham group and TBI group. **(I)** Quantification of DR5-positive cells in sham group and TBI group. Scale bar = 50 μm; *N* = 6 per group. Data are represented as mean ± SD. *p < 0.05 vs. sham; ns, no significance vs. sham. One-way ANOVA, Tukey’s *post hoc* *test*.

The TBI model was induced by controlled cortical impact (CCI) as previously described (Wu et al., 2016; Figure 2A). The rats were anesthetized with 50 mg/kg 1% pentobarbital sodium *via* intraperitoneal injection. The head of the rat was mounted on a stereotaxic frame by ear bars and an incisor bar. Pneumatic brain trauma was induced with a PinPointTM Precision Cortical Impactor (Cary, NC, USA) perpendicular to the intact dura (impactor diameter: 4 mm, impact velocity: 3 m/s, impact duration: 120 ms, brain displacement: 2.5 mm). After trauma, the skull injury site was immediately replaced and sealed with bone wax (ETHICON, Bridgewater, NJ, USA), and the scalp wound was then sutured. Sham-operated animals received the same surgical procedures without CCI.

We also used a standard mechanical injury with hypoxia model to simulate *in vitro* TBI within cells. Briefly, a sterile 21-gauge needle was used to draw parallel scratches across the circular wells of culture plates, 12 scratches in six-well plates and eight scratches in 24-well plates, respectively. Scratch injury may activate neuron death first at the wound edge, later expanding to the entire neuron monolayer (Mori et al., 2002). After the mechanical injury, cell cultures were then cultured in oxygen-deprivation (OGD) conditions (O_2_ was replaced with N_2_) for 6 h to mimic hypoxia (Zhong et al., 2017).

### Drug Administration

sDR5 protein (Sino Biological, China, 10465-H08H) was used *in vivo* to block TRAIL-induced apoptosis (Cui et al., 2010). We injected 10 μg and 25 μg sDR5 protein (10 and 25 μg/rat diluted with 10 μl PBS) intracerebroventricularly using a microinfusion pump at a rate of 3 μl/min, at 30 min and 36 h after TBI. HIF-1α inhibitor, 2ME (Selleck, USA), and activator, DMOG (Selleck, USA), were dissolved in dimethylsulfoxide (DMSO). DMOG (20 mg/kg; Sen and Sen, 2016) and 2ME (20 mg/kg; Schaible et al., 2014) were injected intraperitoneally 30 min after trauma.

For the *in vitro* portion of the experiment, HIF-1α siRNA or scramble siRNA (Genomeditech, Shanghai, China) were mixed with transfection reagent LIPO2000 (Thermo Fisher, Waltham, MA, USA) and delivered 48 h before cell injury. DcR1 peptide (Abcam, Cambridge, MA, USA, ab7880) was delivered as a blocking antibody at different concentrations (0.1 μg/ml, 1 μg/ml, 5 μg/ml, and 10 μg/ml) to inhibit the function of DcR1 0.5 h after mechanical injury and before hypoxia treatment. Additionally, 1 μg/ml recombinant mouse TRAIL protein (Sino Biological, China, aa 118–291) was delivered 16 h after cell injury (Kichev et al., 2014).

### Neurobehavioral Function Assessment

The modified Garcia test and Beam Balance test (Garcia et al., 1995; Rui et al., 2019) were used to evaluate the neurological deficits of animals after TBI and drug treatment. The neurological functions of each group were double-blindly evaluated at 72 h after TBI. The modified Garcia test score was used to test the response capacity, alertness, coordination, and motor skills, which included seven parameters (spontaneous activity, body proprioception, vibrissae touch, spontaneous movement of limbs, lateral turning, forelimb walking, and climbing wall of cage). Each part was assigned three points, for a total of 21 points (Garcia et al., 1995). Beam balance was used to test complex movements and coordination. Rats were placed on a beam to detect their ability to walk and balance. The score ranged from 0 to 4 and was decided according to the distance walked (Rui et al., 2019).

### Brain Water Content

We used the wet–dry method (Lu et al., 2019) to evaluate the brain water content (degree of brain edema) at 72 h after TBI. After anesthesia, the brains of the sacrificed rats were immediately collected and weighed (wet weight). Next, the brains were dried at 100°C for 48 h and weighed again (dry weight). The brain water content was calculated with the following formula: [(wet weight − dry weight)/(wet weight)] × 100% (Lu et al., 2019).

### Western Blot Analysis

Western blot was performed with the same procedure as in the previous study (Li et al., 2013). Cells from the injury area of the rat brain samples were collected and lysed in RIPA lysis buffer (Beyotime, Shanghai, China). After determining the protein concentration with BCA protein assay (Thermo Fisher, Waltham, MA, USA), the protein samples (60 μg/μl) were subjected to sodium dodecyl sulfate-polyacrylamide gel electrophoresis (SDS-PAGE) and transferred to a polyvinylidene difluoride filter (PVDF) membrane (Millipore, Burlington, MA, USA). The membrane was then blocked with 5% non-fat milk at room temperature for 1 h and then incubated with the primary antibody at 4°C overnight. The primary antibodies included are as follows: anti-TRAIL antibody (1:1,000, Thermo Fisher, Waltham, MA, USA, PA5-80165), anti-DR5 antibody (1: 500, Thermo Fisher, Waltham, MA, USA, PA1-957), anti-DcR1 antibody (1:1,000, Abcam, Waltham, MA, USA, ab133658), anti-DcR2 antibody (1:1,000, Novus, St Charles, MO, USA, NBP1-76985), anti-HIF-1α antibody (1:500, Abcam, Cambridge, MA, USA, ab2185), anti-caspase-3 antibody (1:1,000, Abcam, Cambridge, MA, USA, ab13847), anti-GAPDH (1:2,000, Abcam, Cambridge, MA, USA, ab181602), β-actin (1:2,000; Santa Cruz Biotechnology, Santa Cruz, CA, USA). Immunoblots were visualized with an imaging system (Bio-Rad Versa Doc, model 4,000) and were analyzed using ImageJ software (ImageJ, Version 1.4 RRID:SCR_003070).

### Immunofluorescence

Rats were sacrificed under deep pentobarbital anesthesia at 72 h after TBI and transcardially perfused with 0.1 mol/L PBS followed by 4% paraformaldehyde (pH 7.4). The brain was immersed in 4% paraformaldehyde for 24 h then successively immersed in serial 15 and 30% sucrose solutions for 2 days. The brain was cut into 9-μm coronal frozen slices and fixed on a slide for fluorescence staining. The brain slices were incubated with 10% normal donkey serum and 0.1% Triton X-100 for 1 h at room temperature, followed with primary antibody at 4°C overnight. The primary antibodies that were utilized are listed as follows: anti-TRAIL antibody (1:200, Thermo Fisher, Waltham, MA, USA, PA5-80165), anti-DR5 antibody (1:200, Thermo Fisher, Waltham, MA, USA, PA1-957), anti-DcR1 antibody (1:500, Abcam, Cambridge, MA, USA, ab133658), anti-HIF-1α antibody (1:200, Abcam, Cambridge, MA, USA, ab2185), anti-Iba-1 (1:500, Abcam ab5076), anti-NeuN (1:500, Abcam, ab177487). On the second day, the cryosections were incubated with secondary antibody and covered with DAPI (Vector Laboratories Inc.). The slides were visualized with a Leica DMi8 fluorescence microscope (Leica Microsystems, Germany) and analyzed using Leica Application Suite software.

### Fluoro-Jade C Staining

FJC staining was used to identify degenerating neurons after acute neuronal distress, as previously described (Li et al., 2017). Coronal sections were cut and stained with FJC (Biosensis, USA) according to the manufacturers’ protocol. First, the sections were immersed in a solution containing 1% sodium hydroxide in 80% alcohol for 5 min and then in 70% alcohol and distilled water, each for 2 min. Second, the sections were transferred into 0.06% potassium permanganate for 10 min and then rinsed in distilled water for 2 min. Third, the sections were stained with a 0.0001% solution of FJC dye dissolved in 0.1% acetic acid vehicle (pH 3.5) for 10 min. Finally, the sections were washed three times, for 1 min each, in distilled water and then stained with DAPI (Sigma, USA). The images were captured by fluorescence microscope. Last, FJC-positive neurons were manually counted in the injured region of six sections per brain at ×100 magnification using ImageJ software.

### Cresyl Violet Staining

CV staining was used to identify the lesion area on ipsilateral cortex after TBI. Coronal sections (12 μm) from 72 h after TBI were collected every 400 μm. A total of 12 consecutive sections were collected from each rat. The region lacking any CV staining was considered contused brain tissue and was analyzed with ImageJ software. The contusion volume was calculated using the following formula (Schaible et al., 2014), which had been used in the previous study: Contusion volume = 0.4 mm * (Area1 + Area2 + … + Area11 + Area12).

### Cell Viability Assay and Cytotoxicity Assay

Cell viability and cytotoxicity assays were utilized to reflect cell death after drug administration. Each individual treatment reflects six replicates for all assays performed on cell cultures in 96-well plates. Cell viability was measured using the CCK-8 cell counting kit (Beyotime, Shanghai, China). According to the manufacturer’s instruction, 20 μl CCK-8 solution was added to 200 μl of cell culture medium and then cultured at 37°C for 2 h. Next, the absorbance was measured at 450 nm. A lactate dehydrogenase (LDH) cytotoxicity test kit (Beyotime, Shanghai, China) was used to measure the cell cytotoxicity. The protocol followed was that after treatment, cells were cultured with 150 μl 10% LDH reagent (diluted by PBS) at 37°C for 1 h and then centrifuged at 400 μg for 5 min. Last, 120 μl supernatant of each well was transferred to a new 96-well plate, and the absorbance was measured at 490 nm.

### Annexin V and PI Staining

Cells were cultured in 12-well plates and were given different treatments. Cells were trypsinized with 0.25% trypsin (without EDTA) for 3 min and centrifuged at 1,000 μg for 5 min and then resuspended with 300 μl binding buffer. Next, 1 μl Annexin V and 1 μl of PI (Becton Dickinson, Franklin Lanes, NJ, USA) were added to the cell suspension and incubated for 30 min at 37°C in the dark. Subsequently, the cells were analyzed by flow cytometry (FACSCalibur; BD Biosciences, San Diego, CA, USA). The cells were first gated based on forward and side scatter. Surviving cells were determined as FITC-/PI-.

### Statistical Analysis

All data are presented as mean ± standard deviation (SD). Data from different groups were compared using one-way ANOVA followed by Tukey’s *post hoc* *test*. The Kruskal–Wallis test was used to compare data with abnormal distributions. Statistical Package for the Social Sciences (SPSS; version 22.0) and GraphPad Prism (version 6.0) software were used for statistical analysis. Statistical significance was defined as *P* < 0.05.

## Results

### TRAIL and Receptors DR5, DcR1, and DcR2 Expression After TBI

The rat TBI model is presented in Figure 2A. The expression levels of TRAIL and receptors DR5, DcR1, and DcR2 in the ipsilateral cortex around the lesion were analyzed at different time points (12 h, 24 h, 72 h, 120 h, and 168 h) after TBI (Figure 2B). TRAIL protein levels were significantly increased at 24 h and continued to 168 h (*P* < 0.05 vs. the sham group; Figure 2C). DR5 protein expression was also significantly higher at 72 h and 120 h compared to the sham group (*P* < 0.05 vs. the sham group; Figure 2D). DcR1 protein expression was slightly increased and DcR2 showed no change after TBI, and no statistical significance was found at the different time points (Figures 2E,F).

Both TRAIL and DR5 protein levels peaked at 72 h after TBI. Therefore, the rats were sacrificed at 72 h for slice freezing. Double immunofluorescence staining was performed to assess locations of TRAIL and DR5 expression. We found that TRAIL protein was expressed in the microglia and DR5 was expressed in the neuron (Figure 2G). Moreover, the number of TRAIL and DR5 positive cells in the ipsilateral cortical lesion were significantly increased 72 h after TBI when compared with the sham group (*P* < 0.05; Figures 2H,I).

### Soluble DR5 Blocks The TRAIL-Induced Apoptosis and Improves The Neurobehavioral Function

**Figure 3:**
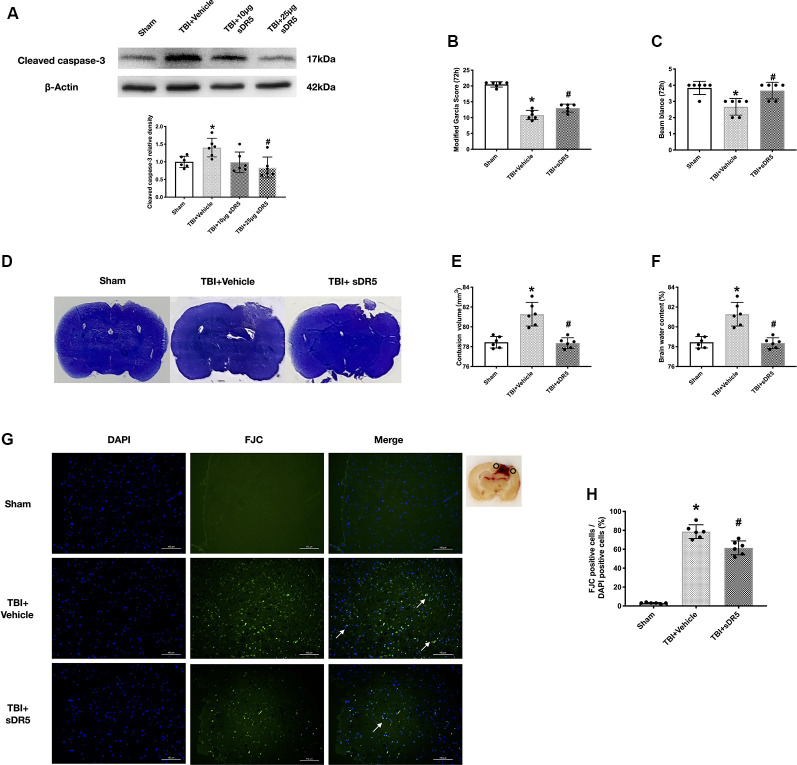
Effects of soluble DR5 (sDR5) administration on neuronal death at 72 h after TBI. **(A)** Representative Western blot images and densitometric quantification of cleaved caspase-3 in different groups treated with vehicle, 10 μg sDR5 and 25 μg sDR5, and sham. **(B)** Modified Garcia score. **(C)** Beam Balance score. **(D)** Representative images of Cresyl Violet (CV) staining. **(E)** Quantification of brain contusion volumes (mm^3^). **(F)** Brain water content. **(G)** Representative images of Fluoro-Jade C (FJC) staining of the peri-contusive cortex. Scale bar = 100 μm. **(H)** Quantification of FJC-positive neurons. Arrow indicates the FJC positive cells. *N* = 6 per group. Data are represented as mean ± SD. *p < 0.05 vs. sham; ^#^*p* < 0.05 vs. TBI + Vehicle. One-way ANOVA, Tukey’s *post hoc* *test*.

To further study the role of the TRAIL signaling pathway in neuronal apoptosis, we used sDR5 intracerebroventricularly to block the effective binding of TRAIL on the neuronal DR5 receptor. The expression of cleaved caspase-3 was detected to explore the effects of different concentrations of sDR5 treatment on TBI-induced neuronal apoptosis. The total of four groups were designed: sham, TBI + vehicle, TBI + 10 μg sDR5, and TBI + 25 μg sDR5. As shown, the expression of cleaved caspase-3 was significantly decreased in the group treated with 25 μg sDR5 compared with the TBI + vehicle group (*P* < 0.05; Figure 3A), but no significant differences were noted between the TBI + 10 μg sDR5 and TBI + vehicle groups. Therefore, we used 25 μg of sDR5 for neurobehavioral function assessment, FJC staining, and quantification of brain water.

The neurological score, lesion area, and brain edema in the ipsilateral cortex were significantly decreased by 25-μg sDR5 treatment compared to the sham group (*P* < 0.05; Figures 3B–F), which indicated that sDR5 treatment improved neurobehavioral function and attenuated secondary injury after TBI. Similarly, when stained by FJC regent, the sDR5 group had fewer FJC-positive cells compared to the sham group in the cortex of the impaired hemisphere (*P* < 0.05; Figures 3G,H), indicating decreased cell death in the ipsilateral cortex after TBI.

### Cellular Location of HIF-1α After Traumatic Brain Injury

**Figure 4:**
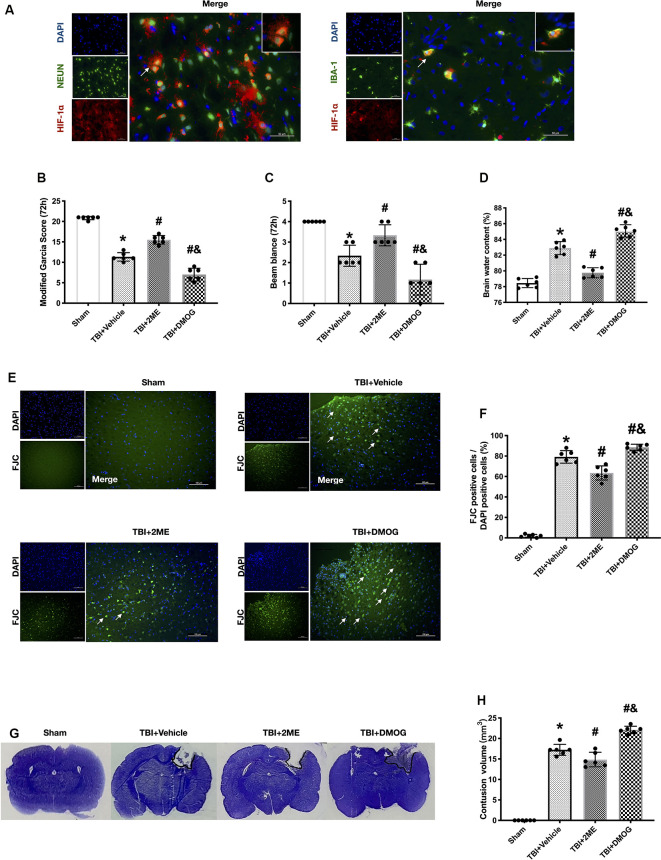
Effects of 2ME and DMOG administration on neuronal death at 72 h after TBI. **(A)** Representative microphotographs of immunofluorescence staining showing localization of Hypoxia factor-1α (HIF-1α; red), Iba-1, and NeuN (green) in injured cerebral cortex after TBI. Scale bar = 50 μm. **(B)** Modified Garcia score. **(C)** Beam Balance score. **(D)** Brain water content. **(E)** Representative images of FJC staining of the peri-contusive cortex. Scale bar = 100 μm. Arrow indicates the FJC positive cells. **(F)** Quantification of FJC-positive neurons. **(G)** Representative images of CV staining. **(H)** Brain contusion volumes (mm^3^). *N* = 6 per group. Data are represented as mean ± SD. *p < 0.05 vs. sham; ^#^*p* < 0.05 vs. TBI + Vehicle; ^&^*p* < 0.05 vs. TBI + 2ME. One-way ANOVA, Tukey’s *post hoc* *test*.

We used double immunofluorescence staining of HIF-1α with NeuN or Iba-1 in the impaired hemisphere of the cerebral cortex. The results showed that HIF-1α was mainly expressed in neuron. Besides, a small amount of HIF-1α was expressed in microglia (Figure 4A). Thus, we continued to study the effects of HIF-1α on TRAIL and related receptors.

### HIF-1α Mediated TRAIL-Induced Apoptosis *in vivo*

**Figure 5:**
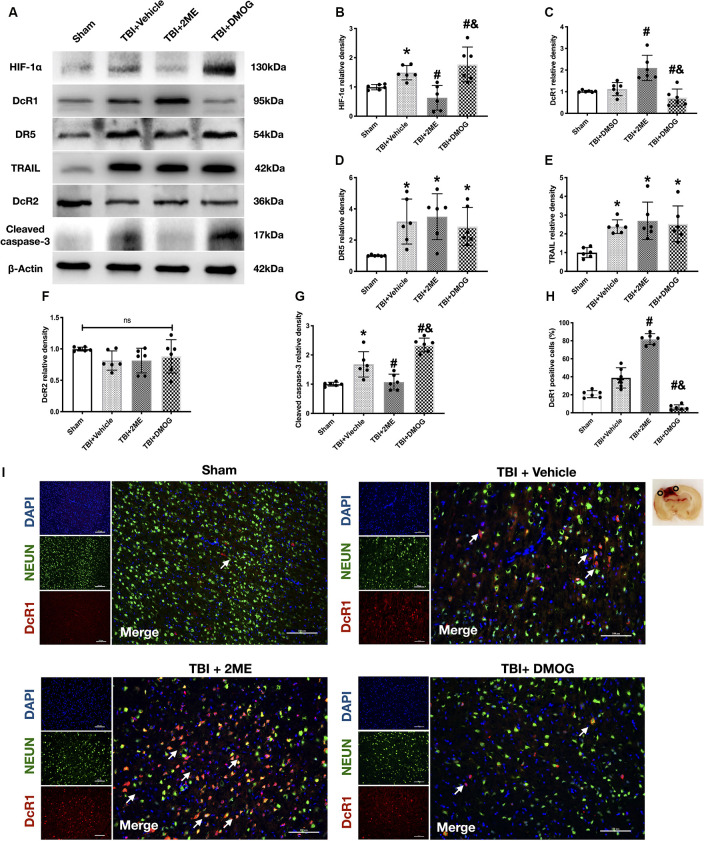
Effects of 2ME and DMOG administration on TRAIL pathway and neuronal apoptosis 72 h after TBI. **(A)** Representative Western blot images. **(B)** Densitometric quantification of HIF-1α. **(C)** Densitometric quantification of DcR1. **(D)** Densitometric quantification of DR5. **(E)** Densitometric quantification of TRAIL. **(F)** Densitometric quantification of DcR2. **(G)** Densitometric quantification of cleaved caspase-3. **(H)** Quantification of DcR1-positive cells. **(I)** Representative microphotographs of immunofluorescence staining showing localization of DcR1 (red) and NeuN (green) in injured cerebral cortex after TBI. Scale bar = 100 μm. Arrow indicates the DcR1 positive cell. *p < 0.05 vs. sham; ^#^*p* < 0.05 vs. TBI + Vehicle; ^&^*p* < 0.05 vs. TBI + 2ME; ns, no significance vs. sham. *N* = 6 per group. Data are represented as mean ± SD. *p < 0.05 vs. TBI + Vehicle. One-way ANOVA, Tukey’s *post hoc* *test*.

The expression of HIF-1α increased after TBI. To further regulate the expression of HIF-1α, TBI rats were treated with the HIF-1α inhibitor, 2ME, and the HIF-1α activator, DMOG. A total of four groups were designed: sham, TBI + vehicle, TBI + 2ME, and TBI + DMOG. The HIF-1α expression was significantly inhibited by 2ME and activated by DMOG compared with the other groups (both *P* < 0.05; Figures 5A,B). The neurobehavioral function, quantified by the modified Garcia score and Beam Balance score, was significantly increased by 2ME treatment and decreased by DMOG treatment compared to the vehicle group (*P* < 0.05; Figures 4B,C). The brain water content was significantly lower in the 2ME group and higher in the DMOG group compared to the vehicle group (*P* < 0.05; Figure 4D). The region of contused brain tissue was defined as the region lacking CV staining. As shown in Figure 4G, the DMOG group has the largest contusion areas and the 2ME group has the smallest contusion areas among the TBI groups (*P* < 0.05; Figure 4H).

Meanwhile, to confirm the role of HIF-1α in neuronal apoptosis, the protein levels of cleaved caspase-3 and the FJC staining results were quantified. The protein levels of cleaved caspase-3 were upregulated in the TBI group when compared with the sham group (*P* < 0.05), while 2ME treatment markedly diminished cleaved caspase-3 expression after TBI (*P* < 0.05). And the DMOG treatment increased the cleaved caspase-3 expression after TBI (*P* < 0.05; Figures 5A–G). The FJC staining showed that there are more FJC-positive cells after TBI compared to the sham group (*P* < 0.05). In the groups that suffered from TBI, 2ME treatment significantly decreased abundance of FJC-positive cells compared to the TBI + vehicle group (*P* < 0.05), and DMOG treatment significantly increased the abundance of FJC-positive cells compared to the TBI + vehicle group (*P* < 0.05; Figures 4E,F).

### HIF-1α Regulated The DcR1 Expression *in vivo*

In investigating the role of HIF-1α in TRAIL-induced neuronal apoptosis, we found that the expression of DcR1 was significantly increased by 2ME administration and decreased by DMOG compared to the vehicle-treated group (both *P* < 0.05; Figures 5A–C). However, no significant difference was found in the expressions of TRAIL and of receptors DR5 and DcR2 after HIF-1α intervention (*P* > 0.05; Figures 5D–F). By using double immunofluorescence staining of DcR1 with NeuN in the impaired hemisphere of the cerebral cortex, we found that neuronal DcR1 was slightly increased after TBI (no significance) and decreased (increased) by 2ME (DMOG) treatment (Figures 5H,I). As DcR1 was a decoy receptor of TRAIL and indirectly inhibited the TRAIL-induced apoptosis by competitively combining with TRAIL, we supposed that HIF-1α mediated TRAIL-induced apoptosis by regulating the expression of DcR1.

### HIF-1α Mediated TRAIL-Induced Apoptosis by Regulating Expression of DcR1 *in vitro*

**Figure 6:**
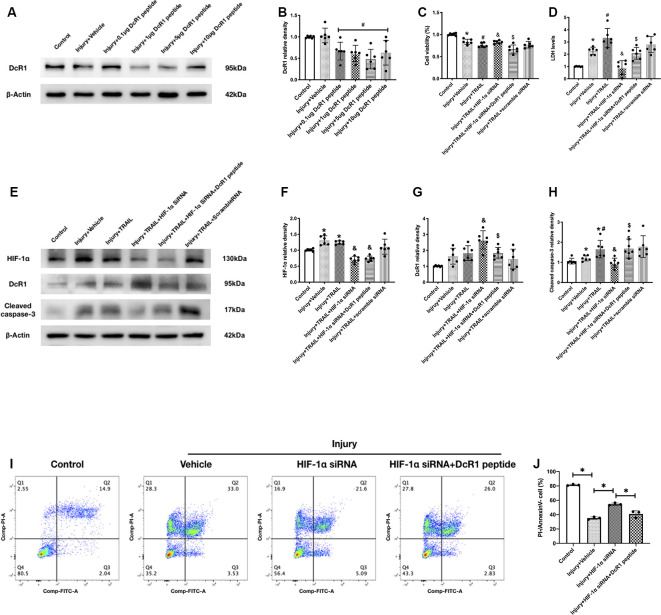
Effect of HIF-1α siRNA TRAIL-induced neuronal apoptosis *in vitro*. **(A)** Representative Western blot images of different DcR1 peptide dosage groups. **(B)** Densitometric quantification of DcR1 of different DcR1 peptide dosage groups. **(C)** Cell viability after siRNA or peptide treatment. **(D)** Cell total lactate dehydrogenase (LDH) level after siRNA or peptide treatment. **(E)** Representative Western blot images of HIF-1α, DcR1, and cleaved caspase-3 after different treatments. **(F)** Densitometric quantification of HIF-1α. **(G)** Densitometric quantification of DcR1. **(H)** Densitometric quantification of cleaved caspase-3. **(I)** Flow-cytometric analysis of HT22 neuronal cells exposed to siRNA or peptide treatment. A surviving cell was defined as PI-/FITC-. **(J)** Statistical analysis of cell survival in different groups. *N* = 3–6 per group. Data are represented as mean ± SD. *p < 0.05 vs. Control; ^#^*p* < 0.05 vs. Injury + Vehicle; ^&^*p* < 0.05 vs. Injury + TRAIL; ^\textdollar^*p* < 0.05 vs. Injury + TRAIL + HIF-1α siRNA. One-way ANOVA, Tukey’s *post hoc* *test*.

We first validated the blocking effect of DcR1 peptide at different concentrations. Six groups were categorized as follows: control, injury + vehicle, injury + 0.1 μg DcR1 peptide, TBI + 1 μg DcR1 peptide, 5 μg DcR1 peptide, and 10 μg DcR1 peptide. Both dosages showed a significant decrease in the protein levels of DcR1 compared to the injury group (*P* < 0.05; Figures 6A,B). Due to the 5-μg DcR1 peptide treatment group having the lowest statistical significance (*P* = 0.0006), 5 μg of DcR1 peptide was applied in the subsequent experiment.

To confirm that HIF-1α mediated TRAIL-induced apoptosis by regulating the expression of DcR1, the cells were divided into the following six groups: control, injury + vehicle, injury + TRAIL, injury + TRAIL + HIF-1α siRNA, injury + TRAIL + HIF-1α siRNA + DcR1 peptide, and injury + TRAIL + scramble siRNA. First, the expression of HIF-1α increased in both the injury + vehicle and injury + TRAIL groups. HIF-1α siRNA significantly decreased the expression of HIF-1α compared to the injury + TRAIL group (*P* < 0.05; Figures 6E,F). Meanwhile, inhibition of HIF-1α expression significantly increased DcR1 expression (Figures 6E–G). Inhibition of HIF-1α increased the cell viability as quantified by CCK-8 and decreased the LDH level (Figures 6C,D) and the production of cleaved caspase-3 (Figure 6H; all *P* < 0.05). The neurons stained with Annexin V and PI (analyzed by flow cytometry) showed that the surviving population (defined as Annexin V-/PI-) decreased after injury and that this was attenuated by HIF-1α siRNA (Figures 6I,J).

The neuroprotective effect of HIF-1α siRNA was significantly abolished by DcR1 peptide according to the results for cleaved caspase-3 expression, cell viability, cytotoxicity, and Annexin V/PI staining, which indicated that the inhibition of HIF-1α expression protected the neurons *via* upregulation of DcR1 (Figures 6C–J; *P* < 0.05).

## Discussion

**Figure 7:**
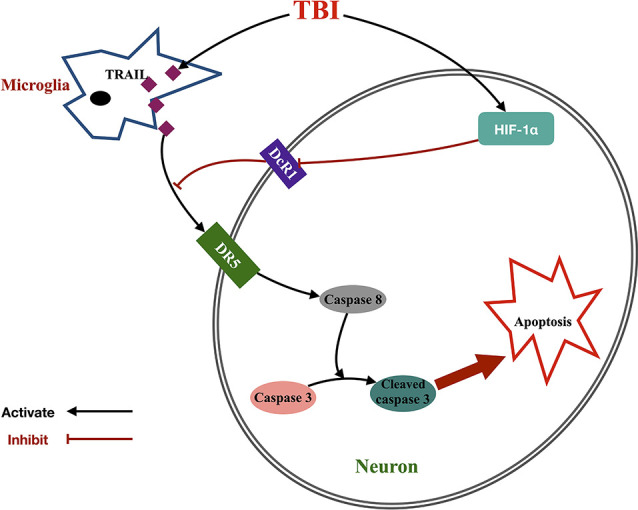
Proposed signaling pathway underlying the effect of HIF-1α-mediated TRAIL-induced neuronal apoptosis after TBI. Microglia increased TRAIL expression after TBI, which activated neuronal DR5 receptor and initiated caspase cascade to apoptosis. Meanwhile, TBI also upregulated HIF-1α expression and further inhibited DcR1 expression. This indirectly increased DR5 function by dismissing the competitive effect of DcR1 to DR5 and then promoted TRAIL-induced neuronal apoptosis.

Apoptosis, also known as programmed cell death, is a major contributor in the pathophysiology of the nervous system and is initiated by extrinsic death ligands or intrinsic stimuli (Green et al., 2014). TRAIL is a death ligand belonging to the TNF superfamily that has been studied and evaluated for its anti-cancer activity (Tisato et al., 2016). In this study, we established that the TRAIL/DR5 signaling pathway played an important role in neuronal death after TBI in rats. Meanwhile, we also found that HIF-1α induced TRAIL-induced apoptosis *via* increasing TRAIL decoy receptor DcR1 expression (Figure 7). In detail, the main findings were as follows: (1) the expression of TRAIL and DR5 in the cortex around the injury was upregulated 12 h and peaked at 72 h after TBI in rats. The increasing trend lasted until 7 days after TBI. Microglia-located TRAIL may be secreted to activate the DR5 receptor found on neurons; (2) functional blockade of TRAIL by the administration of sDR5 successfully attenuated neuronal cell death, brain edema, and injury area while improving neurological behavior in rats; (3) the HIF-1α inhibitor 2ME could also attenuate neuronal cell death, brain edema, and injury area and improve neurological behavior in rats after TBI. Conversely, the HIF-1α agonist, DMOG, presented the opposite result; (4) 2ME (DOMG) treatment increased (decreased) DcR1 expression, with no change in DR5 and DcR2 expression; and (5) HIF-1α siRNA prevented cell death *via* increased expression of DcR1 in the HT-22 neuronal cell line.

Recently, the TRAIL/DR5 pathway has been widely reported to be involved in neuronal apoptosis after ischemia stroke *in vivo* (Martin-Villalba et al., 1999; Cui et al., 2010; Huang et al., 2011; Cantarella et al., 2014). In the hypoxia-ischemia-treated mouse model, it was found that the expressions of TRAIL and DR5 were significantly upregulated after insult (Cui et al., 2010; Huang et al., 2011; Cantarella et al., 2014). The neuronal death may be triggered by the binding of microglia-secreted TRAIL and the neuronal DR5 receptor (Cui et al., 2010). Secondary injury after TBI is the main factor affecting the prognosis of patients. The secondary injury is primarily a pathological process including cellular hyperexcitability, vasogenic and cytotoxic edema, hypoxic-ischemia, oxidative stress, and inflammation, all of which share similarities with the pathological changes after cerebral ischemia (Karve et al., 2016). Therefore, we hypothesize that the TRAIL/DR5 pathway is involved in the pathological change after TBI. Similarly, the expression and upregulation of both TRAIL and DR5 have been identified after TBI in our study, and the cellular localization of TRAIL and DR5 is consistent with previous studies (Cui et al., 2010). Neuroprotection through the blockage of TRAIL by sDR5 further confirmed the involvement of TRAIL/DR5 in neuron apoptosis after TBI.

HIF is a nuclear transcription factor characterized as the master regulator of cellular oxygen homeostasis. It widely participates the tissue survival process by regulating expresion of several key enzymes in cell metabolism glucose transporter (GLUT), angiogenesis (VEGF, VEGFR1, angiopoietin), and free radical scavenging (heme hydroxylase-1; HO-1); Schumacker, 2005; Di Cesare Mannelli et al., 2018). As one of the subunits of HIF, the expression of HIF-1α is largely dependent on oxygen levels. HIF-1α expression is rapidly upregulated in response to the acute phase in secondary injury after TBI (Ding et al., 2009; Li et al., 2013) and is rapidly degraded upon reperfusion and the chronic phase of TBI (Khan et al., 2017). Remarkably, as the HIF-1α pathway is involved in both pathological and neurorepair mechanisms and functions following TBI, the role of HIF-1α in TBI remains relatively controversial (Khan et al., 2017). Under hypoxic conditions after TBI, HIF-1α could have detrimental effects on the blood-brain barrier, brain edema, and apoptosis (Althaus et al., 2006; Ding et al., 2009). The HIF-1α inhibitor, 2ME, was also proven to be neuroprotective 24 h following TBI in mice; however, the detailed mechanism has not been studied (Shenaq et al., 2012; Schaible et al., 2014). In agreement with former research, our study showed the 2ME successfully inhibited HIF-1α expression and provided neuroprotection *via* decreasing neuronal death after TBI *in vivo* and *in vitro*. Additionally, HIF-1α stabilizers/inducers appear to have a different effect than did 2ME. Conversely, HIF-1α induces transcription of EPO and VEGF, which promotes cell survival *via* erythropoiesis, angiogenesis, and anti-apoptosis under mild hypoxia or normoxia (Fan et al., 2009; Wittko-Schneider et al., 2013; Di Cesare Mannelli et al., 2018). Furthermore, VEGF receptors 1 and 2 seem to have diverse effects on central nervous system development and homeostasis (Wittko-Schneider et al., 2013). Interestingly, another study found that treatment with DMOG could reduce both neuronal and cell death after 2 weeks following TBI in mice (Sen and Sen, 2016). This indicates that 2 weeks may be a relatively mild hypoxia or normoxia time point for mice after TBI.

As mentioned before, HIF-1α participates in the regulation mechanism of apoptosis; however, the effect was also dual after TBI (Greijer and van der Wall, 2004). As the promoter of apoptosis, HIF-1α induces transcriptional activation of tumor suppressor proteins BNIP3 and p53, which induce cell apoptosis in the acute phase (Chen et al., 2003; Aminova et al., 2008; Fan et al., 2009). Moreover, it was found that cells with consecutive expression of HIF-1α may be more resistant to apoptosis than normal cells and that the potential anti-apoptotic mechanism may include increased anti-apoptotic factors, EPO, and anerobic metabolism (Akakura et al., 2001; Bianciardi et al., 2006). In this study, we found that HIF-1α may mediate DcR1 expression both *in vivo* and *in vitro* after TBI. Downregulating (upregulating) HIF-1α with 2ME (DMOG) increased the DcR1 level in rats after TBI, and inhibition of HIF-1α by HIF-1α siRNA also increased DcR1 levels in the HT-22 neuron cell line. As reported, overexpression of DcR1 or DcR2 blocks apoptotic signaling of TRAIL (Muzio, 1998). DcR1 inhibits apoptosis by competitive binding to TRAIL (Merino et al., 2006), while DcR2 inhibits apoptosis *via* the formation of heterocomplexes with DR5 to block the subsequent caspase cascade (Clancy et al., 2005; Merino et al., 2006). Furthermore, the expressions of these two decoy receptors of TRAIL have been suggested to confer neuronal protection after ischemic preconditioning in rats (Panneerselvam et al., 2011; Cantarella et al., 2014). In our *in vivo* model, cleaved caspase-3 and cell death were significantly increased after blocking DcR1 activity *via* DcR1 peptide administration. Taken together, we determined that HIF-1α may prompt neuron apoptosis in the injured cortex in the first 3 days after TBI *via* inhibition of DcR1 expression.

Interestingly, a previous study found that HIF-1α regulates the transcription of DcR2 in human colon cancer cell lines (Pei et al., 2010). HIF-1α inactivation increased TRAIL sensitivity in hypoxia-induced TRAIL-resistant tumor cells (Jeong et al., 2010). Additionally, some reports suggested that severe hypoxia leads to p53 activation; however, it was found that neither p53 nor NF-κB contributed to the regulation in this mechanism (Pei et al., 2010). Thus, we hypothesize that the TRAIL decoy receptors DcR1 and DcR2 act in different capacities in different pathological processes. The ratio between decoy receptor (DcR1/2) and death receptor (DR4/5) mediates cell death in different diseases.

It should be mentioned that this study has some limitations. First, though we have identified altered expression of DcR1 *in vivo* under HIF-1α agents, we did not further investigate the function of DcR1 in TRAIL-induced apoptosis in the *in vivo* model. Second, we only used cell lines for investigating the effect of HIF-1α *in vitro*, and there would be some subtle differences between cell lines and primary cells. Third, the pathophysiology after TBI is a complicated process, and the standard *in vivo* mechanical injury with hypoxia model cannot fully represent the changes after TBI. There would also be other mechanisms that contribute to the changes in HIF-1α-mediated apoptosis. And as we mentioned previously, different oxygen metabolism levels and brain regions at different time points might be the reason for the controversial effects of HIF-1α after TBI. It is necessary to study the natural oxygen changes and artificial oxygen intervention after TBI in the future. Fourth, p53 and NF-κB also participated in HIF-1α-mediated apoptosis, and it is still unclear how HIF-1α inhibited DcR1 expression; this part also needs further study.

In conclusion, this study demonstrated that the TRAIL/DR5 signaling pathway induced apoptosis in neurons after TBI. Inhibition of HIF-1α attenuated neuronal death, the insult area, and brain edema but improved neurological function *via* upregulation of expression of TRAIL decoy receptor DcR1, which indirectly reduced apoptosis after TBI. Our results imply that HIF-1α may be an important regulator acting on the TRAIL/DR5 apoptosis signaling pathway following TBI.

## Data Availability Statement

The raw data supporting the conclusions of this article will be made available by the authors, without undue reservation, to any qualified researcher.

## Ethics Statement

The animal protocol was reviewed and approved by the Institutional Ethics Committee of the Second Affiliated Hospital, Zhejiang University School of Medicine. The procedures were conducted according to the National Institutes of Health’s Guide for the Care and the Use of Laboratory Animals and the ARRIVE (Animal Research: Reporting *in vivo* Experiments) guidelines.

## Author Contributions

JZha and YH: conceptualization. YF: methodology and software. JL, XW, and SM: validation. HW and JZhe: formal analysis. YF: investigation. JZha: resources, supervision and project administration. JL and SX: data curation. YF: writing—original draft preparation. YF and CL: writing—review & editing. SC: visualization. YH and JZha: funding acquisition.

## Conflict of Interest

The authors declare that the research was conducted in the absence of any commercial or financial relationships that could be construed as a potential conflict of interest.

## References

[B1] Akakura N. Kobayashi M. Horiuchi I. Suzuki A. Wang J. Chen J.. (2001). Constitutive expression of hypoxia-inducible factor-1α renders pancreatic cancer cells resistant to apoptosis induced by hypoxia and nutrient deprivation. Cancer Res. 61, 6548–6554. 11522653

[B2] Althaus J. Bernaudin M. Petit E. Toutain J. Touzani O. Rami A. (2006). Expression of the gene encoding the pro-apoptotic BNIP3 protein and stimulation of hypoxia-inducible factor-1α (HIF-1α) protein following focal cerebral ischemia in rats. Neurochem. Int. 48, 687–695. 10.1016/j.neuint.2005.12.008 16464515

[B3] Aminova L. R. Siddiq A. Ratan R. R. (2008). Antioxidants, HIF prolyl hydroxylase inhibitors or short interfering RNAs to BNIP3 or PUMA, can prevent prodeath effects of the transcriptional activator, HIF-1α, in a mouse hippocampal neuronal line. Antioxid. Redox Signal. 10, 1989–1998. 10.1089/ars.2008.2039 18774900 PMC2612757

[B4] Bianciardi P. Fantacci M. Caretti A. Ronchi R. Milano G. Morel S.. (2006). Chronic *in vivo* hypoxia in various organs: hypoxia-inducible factor-1α and apoptosis. Biochem. Biophys. Res. Commun. 342, 875–880. 10.1016/j.bbrc.2006.02.042 16596722

[B5] Bossi F. Bernardi S. Zauli G. Secchiero P. Fabris B. (2015). TRAIL modulates the immune system and protects against the development of diabetes. J. Immunol. Res. 2015:680749. 10.1155/2015/680749 25759846 PMC4352427

[B6] Cantarella G. Pignataro G. Di Benedetto G. Anzilotti S. Vinciguerra A. Cuomo O.. (2014). Ischemic tolerance modulates TRAIL expression and its receptors and generates a neuroprotected phenotype. Cell Death Dis. 5:e1331. 10.1038/cddis.2014.286 25032854 PMC4123080

[B7] Carmeliet P. Dor Y. Herbert J. M. Fukumura D. Brusselmans K. Dewerchin M.. (1998). Role of HIF-1α in hypoxia-mediated apoptosis, cell proliferation and tumour angiogenesis. Nature 394, 485–490. 10.1038/28867 9697772

[B8] Chen D. Li M. Luo J. Gu W. (2003). Direct interactions between HIF-1α and Mdm2 modulate p53 function. J. Biol. Chem. 278, 13595–13598. 10.1074/jbc.c200694200 12606552

[B9] Clancy L. Mruk K. Archer K. Woelfel M. Mongkolsapaya J. Screaton G.. (2005). Preligand assembly domain-mediated ligand-independent association between TRAIL receptor 4 (TR4) and TR2 regulates TRAIL-induced apoptosis. Proc. Natl. Acad. Sci. U S A 102, 18099–18104. 10.1073/pnas.0507329102 16319225 PMC1312398

[B10] Cui M. Wang L. Liang X. Ma X. Liu Y. Yang M.. (2010). Blocking TRAIL-DR5 signaling with soluble DR5 reduces delayed neuronal damage after transient global cerebral ischemia. Neurobiol. Dis. 39, 138–147. 10.1016/j.nbd.2010.03.018 20359534

[B11] Dessein P. H. Lopez-Mejias R. Ubilla B. Genre F. Corrales A. Hernandez J. L.. (2015). TNF-related apoptosis-inducing ligand cardiovascular disease in rheumatoid arthritis. Clin. Exp. Rheumatol. 33, 491–497. 25962765

[B12] Di Cesare Mannelli L. Tenci B. Micheli L. Vona A. Corti F. Zanardelli M.. (2018). Adipose-derived stem cells decrease pain in a rat model of oxaliplatin-induced neuropathy: role of VEGF-A modulation. Neuropharmacology 131, 166–175. 10.1016/j.neuropharm.2017.12.020 29241656

[B13] Ding J. Y. Kreipke C. W. Schafer P. Schafer S. Speirs S. L. Rafols J. A. (2009). Synapse loss regulated by matrix metalloproteinases in traumatic brain injury is associated with hypoxia inducible factor-1α expression. Brain Res. 1268, 125–134. 10.1016/j.brainres.2009.02.060 19285046 PMC2668731

[B14] Ding J. Y. Kreipke C. W. Speirs S. L. Schafer P. Schafer S. Rafols J. A. (2009). Hypoxia-inducible factor-1α signaling in aquaporin upregulation after traumatic brain injury. Neurosci. Lett. 453, 68–72. 10.1016/j.neulet.2009.01.077 19429018 PMC2703426

[B15] Erler J. T. Cawthorne C. J. Williams K. J. Koritzinsky M. Wouters B. G. Wilson C.. (2004). Hypoxia-mediated down-regulation of bid and bax in tumors occurs via hypoxia-inducible factor 1-dependent and -independent mechanisms and contributes to drug resistance. Mol. Cell. Biol. 24, 2875–2889. 10.1128/mcb.24.7.2875-2889.2004 15024076 PMC371100

[B16] Fan X. Heijnen C. J. van der Kooij M. A. Groenendaal F. van Bel F. (2009). The role and regulation of hypoxia-inducible factor-1α expression in brain development and neonatal hypoxic-ischemic brain injury. Brain Res. Rev. 62, 99–108. 10.1016/j.brainresrev.2009.09.006 19786048

[B17] Garcia J. H. Wagner S. Liu K. F. Hu X. J. (1995). Neurological deficit and extent of neuronal necrosis attributable to middle cerebral artery occlusion in rats. Statistical validation. Stroke 26, 627–634. 10.1161/01.str.26.4.627 7709410

[B18] Green D. R. Galluzzi L. Kroemer G. (2014). Cell biology. Metabolic control of cell death. Science 345:1250256. 10.1126/science.1250256 25237106 PMC4219413

[B19] Greijer A. E. van der Wall E. (2004). The role of hypoxia inducible factor 1 (HIF-1) in hypoxia induced apoptosis. J. Clin. Pathol. 57, 1009–1014. 10.1136/jcp.2003.015032 15452150 PMC1770458

[B20] Guo Z.-N. Shao A. Tong L.-S. Sun W. Liu J. Yang Y. (2016). The role of nitric oxide and sympathetic control in cerebral autoregulation in the setting of subarachnoid hemorrhage and traumatic brain injury. Mol. Neurobiol. 53, 3606–3615. 10.1007/s12035-015-9308-x 26108186

[B21] Hoffmann O. Zipp F. Weber J. R. (2009). Tumour necrosis factor-related apoptosis-inducing ligand (TRAIL) in central nervous system inflammation. J Mol Med (Berl) 87, 753–763. 10.1007/s00109-009-0484-x 19449143

[B22] Huang Z. Song L. Wang C. Liu J. Q. Chen C. (2011). Hypoxia-ischemia upregulates TRAIL and TRAIL receptors in the immature rat brain. Dev. Neurosci. 33, 519–530. 10.1159/000334475 22286051

[B23] Jeong J. K. Moon M. H. Seo J. S. Seol J. W. Park S. Y. Lee Y. J. (2010). Hypoxia inducing factor-1α regulates tumor necrosis factor-related apoptosis-inducing ligand sensitivity in tumor cells exposed to hypoxia. Biochem. Biophys. Res. Commun. 399, 379–383. 10.1016/j.bbrc.2010.07.082 20659427

[B24] Karve I. P. Taylor J. M. Crack P. J. (2016). The contribution of astrocytes and microglia to traumatic brain injury. Br. J. Pharmacol. 173, 692–702. 10.1111/bph.13125 25752446 PMC4742296

[B25] Khan M. Khan H. Singh I. Singh A. K. (2017). Hypoxia inducible factor-1 α stabilization for regenerative therapy in traumatic brain injury. Neural Regen Res 12, 696–701. 10.4103/1673-5374.206632 28616019 PMC5461600

[B26] Kichev A. Rousset C. I. Baburamani A. A. Levison S. W. Wood T. L. Gressens P.. (2014). Tumor necrosis factor-related apoptosis-inducing ligand (TRAIL) signaling and cell death in the immature central nervous system after hypoxia-ischemia and inflammation. J. Biol. Chem. 289, 9430–9439. 10.1074/jbc.m113.512350 24509861 PMC3979382

[B27] Lawrie A. (2014). The role of the osteoprotegerin/tumor necrosis factor related apoptosis-inducing ligand axis in the pathogenesis of pulmonary arterial hypertension. Vascul. Pharmacol. 63, 114–117. 10.1016/j.vph.2014.10.002 25446166

[B28] Li A. Sun X. Ni Y. Chen X. Guo A. (2013). HIF-1α involves in neuronal apoptosis after traumatic brain injury in adult rats. J. Mol. Neurosci. 51, 1052–1062. 10.1007/s12031-013-0084-7 23979836

[B29] Li Q. Han X. Lan X. Gao Y. Wan J. Durham F.. (2017). Inhibition of neuronal ferroptosis protects hemorrhagic brain. JCI Insight 2:e90777. 10.1172/jci.insight.90777 28405617 PMC5374066

[B30] Lopez-Gomez C. Oliver-Martos B. Pinto-Medel M. J. Suardiaz M. Reyes-Garrido V. Urbaneja P.. (2016). TRAIL and TRAIL receptors splice variants during long-term interferon beta treatment of patients with multiple sclerosis: evaluation as biomarkers for therapeutic response. J. Neurol. Neurosurg. Psychiatry 87, 130–137. 10.1136/jnnp-2014-309932 25736057 PMC4752633

[B31] Lu J. Sun Z. Fang Y. Zheng J. Xu S. Xu W.. (2019). Melatonin suppresses microglial necroptosis by regulating deubiquitinating enzyme a20 after intracerebral hemorrhage. Front. Immunol. 10:1360. 10.3389/fimmu.2019.01360 31258534 PMC6587666

[B32] Martin-Villalba A. Herr I. Jeremias I. Hahne M. Brandt R. Vogel J.. (1999). CD95 ligand (Fas-L/APO-1L) and tumor necrosis factor-related apoptosis-inducing ligand mediate ischemia-induced apoptosis in neurons. J. Neurosci. 19, 3809–3817. 10.1523/JNEUROSCI.19-10-03809.1999 10234013 PMC6782733

[B33] Merino D. Lalaoui N. Morizot A. Schneider P. Solary E. Micheau O. (2006). Differential inhibition of TRAIL-mediated DR5-DISC formation by decoy receptors 1 and 2. Mol. Cell. Biol. 26, 7046–7055. 10.1128/mcb.00520-06 16980609 PMC1592888

[B34] Michowitz Y. Goldstein E. Roth A. Afek A. Abashidze A. Ben Gal Y.. (2005). The involvement of tumor necrosis factor-related apoptosis-inducing ligand (TRAIL) in atherosclerosis. J. Am. Coll. Cardiol. 45, 1018–1024. 10.1016/j.jacc.2004.12.065 15808757

[B35] Mori T. Wang X. Jung J. C. Sumii T. Singhal A. B. Fini M. E.. (2002). Mitogen-activated protein kinase inhibition in traumatic brain injury: *in vitro* and *in vivo* effects. J. Cereb. Blood Flow Metab. 22, 444–452. 10.1097/00004647-200204000-00008 11919515

[B36] Mundt B. Wirth T. Zender L. Waltemathe M. Trautwein C. Manns M. P.. (2005). Tumour necrosis factor related apoptosis inducing ligand (TRAIL) induces hepatic steatosis in viral hepatitis and after alcohol intake. Gut 54, 1590–1596. 10.1136/gut.2004.056929 16227360 PMC1774732

[B37] Muzio M. (1998). Signalling by proteolysis: death receptors induce apoptosis. Int. J. Clin. Lab. Res. 28, 141–147. 10.1007/s005990050035 9801924

[B38] Panneerselvam M. Patel P. M. Roth D. M. Kidd M. W. Chin-Lee B. Head B. P.. (2011). Role of decoy molecules in neuronal ischemic preconditioning. Life Sci. 88, 670–674. 10.1016/j.lfs.2011.02.004 21315738 PMC3070046

[B39] Pei G.-T. Wu C.-W. Lin W.-W. (2010). Hypoxia-induced decoy receptor 2 gene expression is regulated via a hypoxia-inducible factor 1α-mediated mechanism. Biochem. Biophys. Res. Commun. 391, 1274–1279. 10.1016/j.bbrc.2009.12.058 20018172

[B40] Piret J.-P. Minet E. Cosse J.-P. Ninane N. Debacq C. Raes M.. (2005). Hypoxia-inducible factor-1-dependent overexpression of myeloid cell factor-1 protects hypoxic cells against tert-butyl hydroperoxide-induced apoptosis. J. Biol. Chem. 280, 9336–9344. 10.1074/jbc.m411858200 15611089

[B41] Rui Q. Ni H. Lin X. Zhu X. Li D. Liu H.. (2019). Astrocyte-derived fatty acid-binding protein 7 protects blood-brain barrier integrity through a caveolin-1/MMP signaling pathway following traumatic brain injury. Exp. Neurol. 322:113044. 10.1016/j.expneurol.2019.113044 31454490

[B42] Schaible E. V. Windschugl J. Bobkiewicz W. Kaburov Y. Dangel L. Kramer T.. (2014). 2-methoxyestradiol confers neuroprotection and inhibits a maladaptive HIF-1α response after traumatic brain injury in mice. J. Neurochem. 129, 940–954. 10.1111/jnc.12708 24606183

[B43] Schneider P. Olson D. Tardivel A. Browning B. Lugovskoy A. Gong D.. (2003). Identification of a new murine tumor necrosis factor receptor locus that contains two novel murine receptors for tumor necrosis factor-related apoptosis-inducing ligand (TRAIL). J. Biol. Chem. 278, 5444–5454. 10.1074/jbc.M210783200 12466268

[B44] Schumacker P. T. (2005). Hypoxia-inducible factor-1 (HIF-1). Crit. Care Med. 33, S423–S425. 10.1097/01.ccm.0000191716.38566.e0 16340411

[B45] Sen T. Sen N. (2016). Treatment with an activator of hypoxia-inducible factor 1, DMOG provides neuroprotection after traumatic brain injury. Neuropharmacology 107, 79–88. 10.1016/j.neuropharm.2016.03.009 26970014 PMC4912959

[B46] Shenaq M. Kassem H. Peng C. Schafer S. Ding J. Y. Fredrickson V.. (2012). Neuronal damage and functional deficits are ameliorated by inhibition of aquaporin and HIF1α after traumatic brain injury (TBI). J. Neurol. Sci. 323, 134–140. 10.1016/j.jns.2012.08.036 23040263

[B47] Stoica B. A. Faden A. I. (2010). Cell death mechanisms and modulation in traumatic brain injury. Neurotherapeutics 7, 3–12. 10.1016/j.nurt.2009.10.023 20129492 PMC2841970

[B48] Tisato V. Gonelli A. Voltan R. Secchiero P. Zauli G. (2016). Clinical perspectives of TRAIL: insights into central nervous system disorders. Cell. Mol. Life Sci. 73, 2017–2027. 10.1007/s00018-016-2164-7 26910728 PMC4834097

[B49] Wittko-Schneider I. M. Schneider F. T. Plate K. H. (2013). Brain homeostasis: VEGF receptor 1 and 2-two unequal brothers in mind. Cell. Mol. Life Sci. 70, 1705–1725. 10.1007/s00018-013-1279-3 23475067 PMC3632714

[B50] Wu G. S. Burns T. F. Zhan Y. Alnemri E. S. El-Deiry W. S. (1999). Molecular cloning and functional analysis of the mouse homologue of the killer/DR5 tumor necrosis factor-related apoptosis-inducing ligand (TRAIL) death receptor. Cancer Res. 59, 2770–2775. 10383128

[B51] Wu H. Shao A. Zhao M. Chen S. Yu J. Zhou J.. (2016). Melatonin attenuates neuronal apoptosis through up-regulation of K( + ) -Cl(−) cotransporter KCC2 expression following traumatic brain injury in rats. J. Pineal Res. 61, 241–250. 10.1111/jpi.12344 27159133

[B52] Wu Y. Y. Hsu J. L. Wang H. C. Wu S. J. Hong C. J. Cheng I. H. (2015). Alterations of the neuroinflammatory markers IL-6 and TRAIL in alzheimer’s disease. Dement. Geriatr. Cogn. Dis. Extra 5, 424–434. 10.1159/000439214 26675645 PMC4677720

[B53] Zhong J. Jiang L. Huang Z. Zhang H. Cheng C. Liu H.. (2017). The long non-coding RNA Neat1 is an important mediator of the therapeutic effect of bexarotene on traumatic brain injury in mice. Brain Behav. Immun. 65, 183–194. 10.1016/j.bbi.2017.05.001 28483659

[B54] Zhou Y. Shao A. Yao Y. Tu S. Deng Y. Zhang J. (2020). Dual roles of astrocytes in plasticity and reconstruction after traumatic brain injury. Cell Commun. Signal. 18:62. 10.1186/s12964-020-00549-2 32293472 PMC7158016

